# Plasma heme pool compartmentalization is linked to pathophysiology in Sickle Cell Disease

**DOI:** 10.1371/journal.pone.0343527

**Published:** 2026-03-26

**Authors:** Moritz Saxenhofer, Daniel Couto, Sandra Mena Perez, Marlies Illi, Justine Brodard, Linet Njue, Ioannis Chanias, Cesare Medri, Gerasimos Tsilimidos, Nicole Hadorn, Christelle Chirlias, Ana-Patricia Batista Mesquita, Lucas Veuthey, Anna Schnell, Stefanie Graeter, Gregory J. Kato, Jacqueline Adam, Ramona Merki, Emmanuel Levrat, Kaveh Samii, Mila Keiser, Kathrin Susann Bieri, Alan G. Haynes, Anne Angelillo-Scherrer, Lorenzo Alberio, Francesco Grandoni, Mathilde Gavillet, Thomas Gentinetta, Alicia Rovó, Alexander Schaub

**Affiliations:** 1 CSL Behring, CSL Biologics Research Centre, Bern, Switzerland,; 2 Swiss Institute for Translational and Entrepreneurial Medicine, sitem-insel, Bern, Switzerland; 3 Department of Hematology and Central Hematology Laboratory, Inselspital, Bern University Hospital, Bern, Switzerland; 4 Service and Central Laboratory of Haematology, Department of Oncology and Department of Laboratory Medicines and Pathology, Lausanne University Hospital (CHUV) and University of Lausanne (UNIL), Lausanne, Switzerland; 5 CSL Behring, Clinical Research & Development, King of Prussia, Pennsylvania, United States of America; 6 Oncology and Hematology Department, Aarau Cantonal Hospital, Aarau, Switzerland; 7 Department of Hemato-Oncology, Hôpital Cantonal Fribourg, Fribourg, Switzerland; 8 Clinical Hematology Unit, Geneva University Hospitals (HUG), Geneva, Switzerland; 9 University of Bern, Department of Clinical Research, Bern, Switzerland; University of Illinois at Chicago, UNITED STATES OF AMERICA

## Abstract

Heme toxicity plays a central role in the pathophysiology of Sickle Cell Disease (SCD), contributing to severe complications such as vaso-occlusion and acute chest syndrome. The continuous release of hemoglobin and heme from increased intravascular hemolysis can exceed the capacity of protective scavenger proteins, leading to heme accumulation in plasma. Interactions with various binding partners result in the formation of different plasma heme species and the compartmentalization of the plasma heme pool. In an observational biomarker study, we used novel bioanalytical assays to quantify plasma heme species in 36 stable-state SCD patients and 36 age, sex, and ethnicity-matched controls. Our results revealed substantially different compartmentalization of plasma heme, despite similar levels of total plasma heme in SCD patients (50 µmol/L) and controls (43 µmol/L). Using a correlation analysis across 85 biomarkers, we examined the association of specific heme species with SCD pathophysiology. Hemopexin-accessible heme (HAH) emerged as a refined indicator of heme burden linked to pathways driving severe SCD complications. A strong inverse correlation was observed between HAH and hemopexin (R = –0.73, p < 0.001), suggesting that hemopexin deficiency contributes to elevated HAH levels. Accurate characterization of clinically relevant plasma heme species and understanding their effects on SCD pathophysiology is essential for the development of new targeted therapies.

## Introduction

Sickle cell disease (SCD) is a genetic disorder characterized by the presence of abnormal hemoglobin S (HbS), which leads to the deformation of red blood cells (RBCs) into a sickle shape under low oxygen conditions [1,2]. HbS significantly impacts the functionality and lifespan of RBCs, which are prone to rupture and release of cell-free hemoglobin (CFH) into circulation [3]. CFH and its breakdown product heme are potent pro-oxidants that interact with circulating and vascular cells, causing sterile inflammation and promoting vascular complications [4]. The induction of oxidative stress by CFH and heme plays a key role in SCD pathophysiology and phenotypic variability [5]. Under physiological conditions, the high-affinity scavenger proteins haptoglobin (Hp) and hemopexin (Hpx) provide a pathway to efficiently neutralize and eliminate CFH and heme from the circulation via the formation of stable complexes [6–8]. CFH bound to Hp within the haptoglobin-hemoglobin (Hp-Hb) complex is recognized by the CD163 receptor on macrophages, which facilitates its uptake and intracellular degradation within the endosomal system [9,10]. This pathway leads to the degradation of heme via heme oxygenase-1 (HO-1), contributing to the clearance of heme and its pro-oxidant effects. Similarly, Hpx-heme complex is taken up by hepatocytes and macrophages via the CD91 receptor [11], also resulting in heme degradation by HO-1 [12].

In SCD and other hemolytic disorders, the protective capacity of the scavenger-mediated degradation pathways is often exhausted due to the continuous intravascular release of hemolysis products and the immediate consumption of Hp and Hpx [13]. Depletion of these protective scavengers leads to an accumulation of CFH in plasma that is oxidized to methemoglobin, which readily releases heme into the bloodstream [14]. In plasma, heme interacts with various binding partners of relatively low affinity, such as albumin [15], alpha-1-microglobulin [16], and other plasma proteins that act as a reversible heme reservoir and latent source of heme toxicity. Impaired elimination of CFH and heme results in a heterogeneous pool of different heme species, each with different reactivity and toxic potential [4]. While total plasma heme is used as a clinical biomarker of hemolysis [17], it may not appropriately reflect the pathological potential of the various heme species [18,19]. Currently, most commercially available assays assess total plasma heme concentrations and are not specific for measuring individual compartments of the heme pool [14]. Moreover, they may suffer from a variety of false-positive interferences, including from bilirubin, and other limitations that can lead to an overestimation of total plasma heme concentrations [20]. Discrimination and accurate quantiﬁcation of reactive heme species with deleterious oxidative potential (such as CFH and plasma protein-bound heme) versus stabilized heme compartments (such as Hp-Hb and Hpx-heme complexes) will help elucidating the deleterious mechanisms downstream of RCB lysis. Characterizing the compartmentalization of the plasma heme pool in SCD is crucial for understanding plasma heme toxicity, informing clinical decisions, and developing new targeted therapeutic interventions.

In an observational biomarker study comparing steady-state SCD patients and a non-hemolytic control group, we investigated the dynamics between plasma heme compartmentalization and the depletion of the protective scavenger proteins. We quantified different heme compartments in plasma, introducing three novel methodologies to quantify Hp-Hb and Hpx-heme complexes, and Hpx-accessible heme (HAH), representing an unstable plasma heme reservoir. To characterize the toxic potential of the total plasma heme pool in SCD and controls, we calculated the relative distribution of heme among different compartments in both groups. Different heme species in SCD were compared to a comprehensive set of hematological and inflammatory biomarkers to assess the association of individual heme compartments with different pathophysiological processes. Insights into the compartmentalization of the plasma heme pool and the disease mechanisms affected by individual heme species may help to better understand plasma heme toxicity and support the clinical interpretation of total plasma heme in SCD. Our findings are expected to inform the development of novel therapeutic strategies to mitigate the adverse effects of intravascular hemolysis. Ultimately, this research aims to improve the clinical management and quality of life for individuals living with SCD.

## Methods

### Study design

Adult patients (≥18 years) with HbSS or HbS/β‑thalassemia (Sβ⁺ or Sβ⁰) SCD, and a matched control group were enrolled in a cross-sectional observational study in Switzerland. Recruitment took place at the Bern University Hospital (Inselspital) between June 18, 2020, and February 27, 2023, and at the Lausanne University Hospital (CHUV) between August 31, 2021, and August 10, 2022. Healthy volunteers without any hemolytic disorder were selected to match the SCD group in terms of sex, age category (<30 years or ≥30 years), and ethnicity. Blood and urine samples were collected from patients in a stable state during a routine hematology clinic visit and from controls in a dedicated appointment. Current and past (≤2 years) clinical information was collected from the patient’s medical files. The study was approved by the competent ethics committee (Swissethics, BASEC registration number 2022−01350), and written informed consent was obtained from all participants.

### Analysis of laboratory parameters and biomarkers

Clinical chemistry and hematological analyses were performed by the local central laboratories of the hospitals. Laboratory test results were transferred into the central study database (secuTrial) by a study nurse. Whole blood was collected into EDTA-coated tubes to prevent coagulation and hemolysis. Samples were kept on ice and processed within 30 minutes of collection. Plasma was separated by centrifugation at 1,500 × g for 15 minutes at 4°C. Aliquots were stored at −80°C until analysis. Prior to each analysis, plasma samples were thawed at room temperature and centrifuged at 14,000 × g for 5 minutes at 2–8 °C to remove any residual cellular debris.

### Research biomarkers in plasma and urine

Biomarker quantification in plasma and urine was performed using multiplex immunoassays and enzyme-linked immunosorbent assays (ELISAs). A total of 29 plasma proteins and 13 urinary proteins were measured using multiplex panels from R&D Systems (S3 Table and S4 Table). Plasma concentrations of HMGB1 and MD-2 were determined by ELISA using kits from IBL/Tecan and RayBiotech, respectively (S3 Table). Urinary MCP-1 levels were quantified using an ELISA kit from R&D Systems, while N-acetyl-β-D-glucosaminidase (NAG) concentrations were assessed via enzymatic assays using kits from Roche and R&D Systems, respectively (S4 Table). All assays were conducted in accordance with the manufacturers’ protocols.

### Quantification of Hpx and Hp by LC-MS/MS

Calibration curves were prepared using cynomolgus monkey plasma as a surrogate matrix and pooled human plasma for quality control. Human Hp1–1 and Hpx (CSL Behring) were spiked into plasma for standard preparation. Plasma samples (10 µL) were spiked with isotopically labeled internal standards specific for Hp and Hpx (NPANPV^Q and NFP^SPVDAAFR peptides). Proteins were denatured with 1% sodium deoxycholate and ammonium bicarbonate, incubated at 70 °C for 20 min, and digested with trypsin (1 µg/µL) at 37 °C for 1 h. Digestion was quenched with 10% formic acid, centrifuged, and supernatants transferred to LC–MS vials.

Chromatographic separation was performed on an Acquity UPLC M-Class system using a µPAC 50 cm C18 column at 10 µL/min. Mobile phases were 0.1% formic acid in water (A) and acetonitrile (B). Detection was by MRM on a QTRAP 6500 + mass spectrometer in positive ion mode. Transitions for Hp and Hpx peptides and their internal standards were monitored. Quantification was based on peak area ratios using SCIEX OS software. Calibration curves were constructed from response ratios versus known concentrations. LLOQ was 9.94 µg/mL for Hp and 2.73 µg/mL for Hpx.

### Quantification of free Hb and Hp-Hb complexes by HPLC

Plasma samples were analyzed by SEC–HPLC (Ultimate 3000SD, ThermoFisher) with a Diol-300 column (300 × 8 mm, 3 µm) and PBS (pH 7.4) as mobile phase at 1 mL/min. Detection was at 280 nm and 414 nm. Peak areas for Hp–Hb complexes, free Hb, and other heme-binding proteins were quantified by interpolation to a hemoglobin standard curve.

### Quantification of Hpx-Heme complexes

Plasma was diluted 1:4 with PBS and depleted of albumin and IgG using an Affinity Removal System column (Agilent). Depleted samples were injected onto a Diol-300 SEC column under the same conditions as above. Hpx–heme was quantified by integrating the peak at ~9 min retention time, calibrated against a standard curve.

### Quantification of Hpx-Accessible Heme (HAH)

HAH was determined by comparing Hpx–heme levels before and after addition of 100 µM Hpx (plasma-derived, CSL). Plasma was diluted 1:4 with PBS or PBS + Hpx, incubated at 37 °C for 1 h, and analyzed by two-step HPLC (albumin/IgG depletion followed by SEC). Detection was at 414 nm; quantification was based on peak area at ~12.2 min retention time. Each sample was analyzed in paired injections (with and without Hpx), measured in duplicate. Columns were cleaned between runs with PBS + 500 µM Hpx to prevent carryover.

### Characterization of the plasma heme pool

The total concentration of plasma heme was quantified using the colorimetric assay QuantiChrom (BioAssay Systems) according to the manufacturer’s instructions. The median concentration of each heme species was used to derive its relative abundance in the SCD and control plasma heme pool. The relative abundance was determined by calculating the proportion of each heme species in relation to the sum of all quantified heme species. The unspecified fraction of the total plasma heme pool was determined by subtracting the concentrations of CFH, Hp-Hb complex, Hpx-heme complex, and HAH from the total plasma heme concentration.

### Statistical analysis

Linear regression was used to compare SCD and control participants for continuous variables. Models were adjusted for stratifying variables – age (dichotomous), sex, and ethnicity, and p-values were adjusted according to Benjamini & Hochberg to control the false discovery rate. Where model assumptions were violated, variables were log-transformed. To assess monotonic associations between variables, Spearman’s rank correlation analysis was performed for the SCD and control groups separately. Spearman’s rho was selected due to its non-parametric nature and robustness to non-normal distributions and outliers. Pairwise Spearman correlation coefficients and corresponding p-values were computed between individual heme species, total plasma heme concentration, and SCD-related biomarkers. Correlations with a p-value < 0.05 were considered statistically significant. Heatmaps were generated using the pheatmap package in R. Only markers that significantly correlated with at least one heme compound were included in the heatmap. A dendrogram was generated from hierarchical clustering applied to the columns of the correlation matrix, using complete linkage and Euclidean distance.

## Results

### Study population

Between June 2020 and February 2023, 36 patients with SCD and 36 controls were enrolled in the study after giving written informed consent. The median age of participants was 32 in the SCD group and 33 in the control group (Table 1). The proportion of females and participants of African descent was the same in both groups, minimizing the effect of demographic confounders. All patients were under treatment according to the standard of care. 19% of SCD patients had HbS/β-thalassemia genotypes (3 HbS/β^0^ and 4 HbS/β^+^). No patients with HbSC genotype were included. 78% were currently treated with hydroxyurea (S1 Table).

**Table 1:** Demographic characteristics of the study population. Median age in years is shown for each group, with the interquartile range in brackets. For other parameters, the number and percentage of individuals per group are indicated.

Characteristic	Control, N = 36	SCD, N = 36
**Age**	33 (28, 38)	32 (24, 39)
**Gender**		
male	13 (36%)	13 (36%)
female	23 (64%)	23 (64%)
**Ethnicity**		
African descent	31 (86%)	31 (86%)
Mediterranean descent	5 (14%)	3 (8.3%)
Middle Eastern, Asian, and Indian descent	0 (0%)	1 (2.8%)
Other	0 (0%)	1 (2.8%)

### Reactive plasma heme compartments prevail in SCD but not in controls

**Fig 1:**
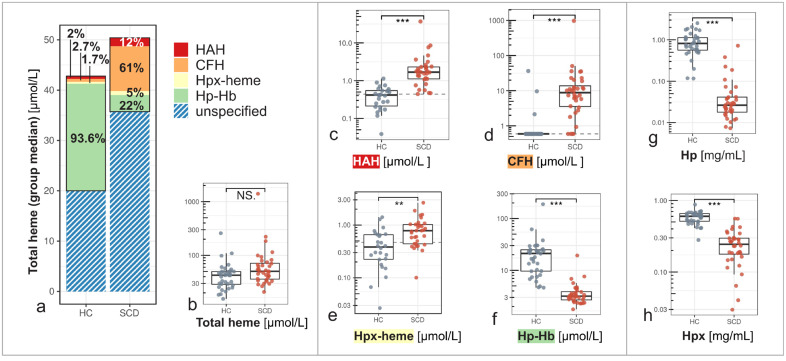
Divergent compartmentalization of the plasma heme pool in SCD patients and controls (HC). **(a)** Stacked bars illustrate median concentrations of different heme species and their relative abundance in the plasma heme pool of healthy controls (HC) and sickle cell disease (SCD) patients. The proportions (in %) of Hpx-accessible heme (HAH; red), cell-free hemoglobin (CFH; orange), Hpx-bound heme (Hpx-heme; yellow), and haptoglobin-hemoglobin (Hp-Hb) complexes (green) are shown in relation to the sum of all quantified heme species. The difference between all quantified heme species and total plasma heme concentrations measured by a colorimetric assay (combined height) is shown as a blue hatched bar (unspecified). **(b)** Total plasma heme concentrations were not significantly different (NS.) in HC and SCD individuals. Plasma concentrations of the reactive heme species **(c)** HAH, **(d)** CFH, and non-reactive complexes **(e)** Hpx-heme, and **(f)** Hp-Hb diverged between groups. Plasma levels of protective scavenger proteins **(g)** Hp and **(h)** Hpx were strongly depleted in SCD. Boxplots show median, interquartile range (IQR), and whiskers (1.5 × IQR). Significant differences between groups are indicated by *** (p < 0.001) and ** (p ≤ 0.01).

**Fig 2:**
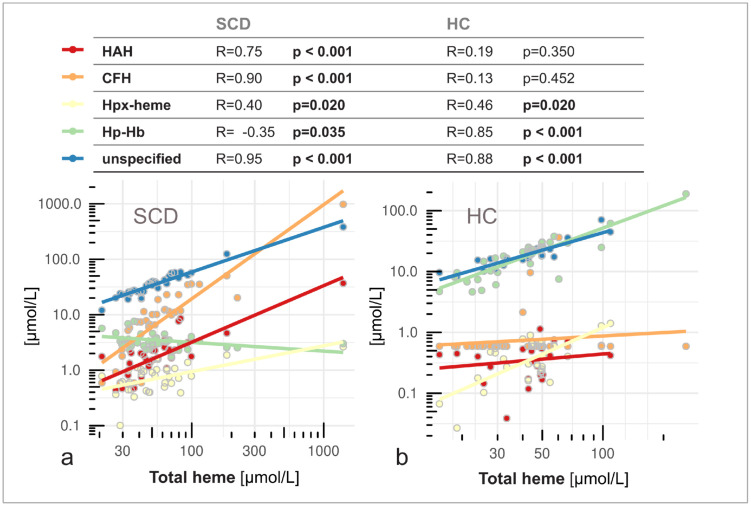
Total heme concentrations were correlated with increased reactive heme species in SCD and non-reactive heme species in controls. **(a)** In SCD, higher levels of total plasma heme were predominantly driven by increased concentrations of reactive HAH (red) and CFH (orange), showing strong positive correlations. Hpx-heme complex (yellow) increased moderately while the concentrations of Hp–Hb complex (green) were lower at higher levels of total heme. **(b)** In controls (HC), total heme correlated strongly with non-reactive Hp–Hb and Hpx–heme complexes but not with HAH or CFH. Unspecified fractions of total heme (blue) correlated similarly with total heme concentrations in both groups. The table shows correlation coefficients (R) and p-values (significant values in bold). Lines indicate linear regression fits for individual heme species.

The proportion of reactive heme compartments was strongly elevated in the plasma heme pool of SCD patients compared to controls (Fig 1a). 73% of the quantified heme pool in SCD consisted of CFH (61%) and HAH (12%) (Fig 1a). In controls, this proportion was only 4.7% (2.7% CFH and 2.0% HAH). The median concentration of HAH was 4 times higher in SCD (1.68 µmol/L) compared to controls (0.42 µmol/L) (p < 0.001) (Fig 1c), and CFH was at least 15 times higher in SCD individuals (9 µmol/L) compared to controls (0.59 µmol/L) (p < 0.001) (Fig 1d). Most controls had CFH levels below the sensitivity limit of the assay (0.59 µmol/L). For these cases, we used values at this lower limit of quantification for the analysis instead. In contrast, the non-reactive heme compartments were highly abundant in controls (95.3%), represented by 93.6% Hp-Hb complex and 1.7% Hpx-heme complex (Fig 1a). In SCD, non-reactive heme compartments accounted for only 27% of the specified heme pool, consisting of 22% Hp-Hb complex and 5% Hpx-heme complex. Importantly, the median concentration of total plasma heme, measured according to an established absorbance-based method, was only slightly higher in SCD (50 µmol/L) compared to controls (43 µmol/L), and not statistically significant (p = 0.06) (Fig 1b). Divergence in the compartmentalization of the plasma heme pool between groups were confirmed by a strong positive correlation between total plasma heme and CFH in SCD (r = 0.90, p < 0.001) but not in controls (r = 0.13, p = 0.45) while total plasma heme correlated positively with Hp-Hb complex in controls (r = 0.85, p < 0.001) but negatively in the SCD group (r = −0.35, p = 0.04) (Fig 2).

Total plasma heme concentrations were higher in patients with the HbSS genotype than in those with S/β-thalassemia (p = 0.016). However, the levels of individual heme species did not differ significantly between SCD genotypes or between hydroxyurea-treated and untreated patients (S1 Fig). To assess potential confounding of the total heme concentration due to spectral interference with non-heme chromophores [20], we correlated the unspecified fraction of the total plasma heme with total bilirubin concentrations. In SCD, the unspecified fraction of total plasma heme was strongly correlated with bilirubin (r = 0.85, p < 0.001) (S2 Fig). This correlation was not significant in controls (r = 0.18, p = 0.39), who had lower total bilirubin levels (S2 Table). However, when the unspecified fraction was normalized using bilirubin concentrations, the correlation with total plasma heme (Fig 2) was lost in both groups (SCD: r = −0.12, p = 0.73; controls: r = 0.38, p = 0.06).

### Impaired scavenger-mediated elimination of reactive heme species

**Fig 3:**
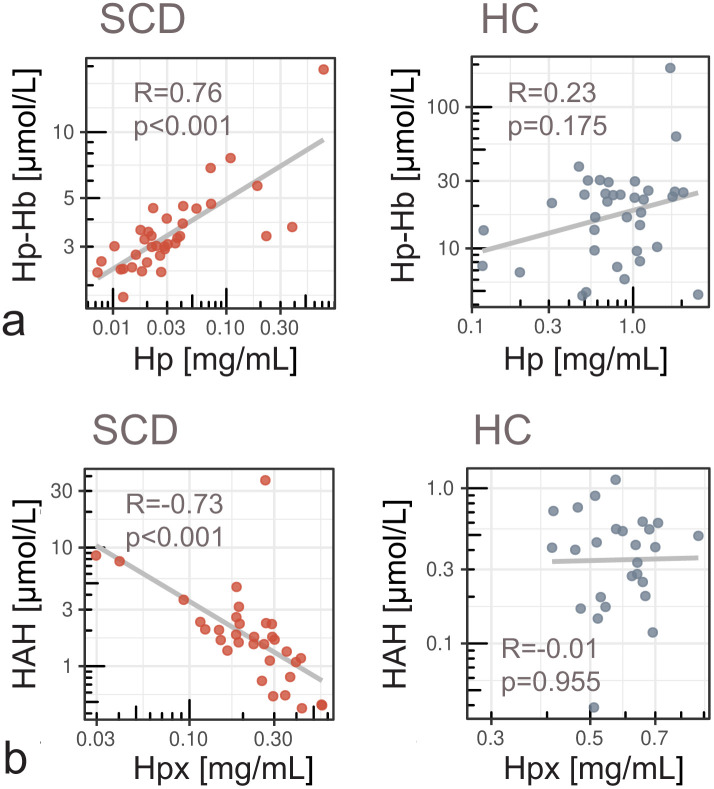
The concentration of individual heme species depends on the availability of Hp and Hpx scavenger proteins. **(a)** The levels of Hp–Hb complex were strongly correlated with Hp concentrations in SCD. In controls (HC), Hp-Hb was not correlated with Hp. **(b)** In SCD, low levels of Hpx were significantly associated with increased levels of Hpx-accessible heme (HAH) but no such correlation was observed in controls. Correlation coefficients and p-values are indicated. Lines show linear regression fits.

The median concentration of Hp, the natural high-affinity scavenger of CFH, was 27-fold lower in SCD (0.03 mg/mL), compared to controls (0.82 mg/mL) (p < 0.001) (Fig 1g). Congruent with this Hp depletion in SCD, the concentration of the neutralizing Hp-Hb complex was 7 times lower in SCD (3 µmol/L) compared to controls (21 µmol/L) (p < 0.001) (Fig 1f). Hp-Hb and Hp were strongly correlated in SCD (r = 0.76, p < 0.001) but not in controls (r = 0.23, p = 0.18) (Fig 3a). The heme-scavenger Hpx was significantly lower in SCD patients (0.24 mg/mL) compared to controls (0.60 mg/mL) (p < 0.001) (Fig 1h). We found a strong negative correlation between Hpx and HAH in SCD (r = −0.73, p < 0.001), but not in controls (r = −0.01, p = 0.96) (Fig 3b). Hpx-bound heme (Hpx-heme complex) was low in both groups, and many values were below the assay’s sensitivity limit. However, Hpx-heme was higher in SCD (0.78 µmol/L) compared to controls (0.39 µmol/L) (p = 0.002) (Fig 1e).

### Pathophysiological signatures of individual heme compartments in SCD

**Fig 4:**
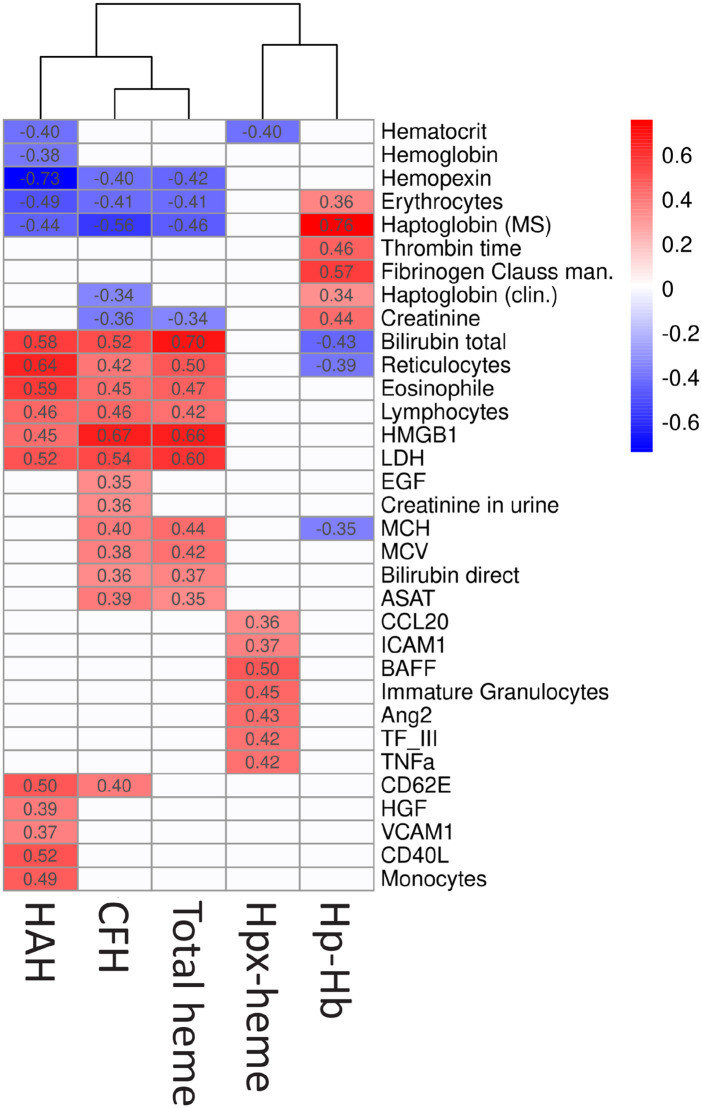
Heme species exhibit divergent correlation patterns with clinical biomarkers in SCD. Spearman correlations were calculated between four heme species (HAH, CFH, Hpx–heme, Hp–Hb), total plasma heme, and 85 SCD-related biomarkers. Numeric values represent pairwise correlation coefficients, and the color gradient indicates the strength and direction of the correlation. Only markers that showed a significant correlation (p < 0.05) with at least one heme species or total heme are shown. Non-significant correlations appear as blank cells. The dendrogram illustrates the similarity between heme species based on their correlation patterns with biomarkers. Full biomarker names are provided in S2–S4 Tables.

Analyses of correlations with 85 hematological and inflammatory biomarkers (S2-S4 Tables) revealed distinct associations between specific heme compartments and the pathophysiology of SCD (Fig 4). We found very similar correlation patterns of CFH and total plasma heme, corroborating the high contribution of CFH to total plasma heme (Fig 1a). Low affinity, labile heme compartments (HAH and CFH) and total plasma heme equivalently showed positive correlations with markers of hemolysis, such as bilirubin, reticulocytes, and LDH, and a negative correlation with Hpx, Hp, and erythrocyte counts. The correlation patterns of high affinity, stable heme compartments (Hpx-heme and Hp-Hb complex) were clearly distinct and, in the case of Hp-Hb, showed inverse associations with hematological biomarkers as compared to labile heme species or total plasma heme. We found each heme compartment to also correlate with distinct groups of biomarkers, which may indicate specific effects of HAH, CFH, Hpx-heme and Hp-Hb complex on the SCD pathophysiology. Biomarkers of endothelial (VCAM1) and platelet activation (sCD40L) were exclusively correlated with HAH. CFH and total plasma heme were associated with erythrocyte markers MCH and MCV, renal markers (EGF and urine creatinine), and further markers of hemolysis (direct bilirubin and aspartate aminotransferase levels). Hpx-heme complex specifically correlated with immune cell (BAFF, CCL20, TNFa) and endothelial (ICAM1) activation markers, as well as coagulation marker TFIII. Hp-Hb complex correlated negatively with total bilirubin and reticulocytes, and positively with Hp, coagulation markers (fibrinogen, thrombin time), and creatinine in plasma.

## Discussion

This study presents the first comprehensive quantification of plasma heme compartments, including the Hp-Hb complex, the Hpx-heme complex, and the HAH reservoir. A comparison with 85 hematological, inflammatory, and hemostatic biomarkers indicated distinct associations of different heme compartments with pathophysiological processes. The insights from this study contribute to a better understanding of the compartmentalization and toxicity of the plasma heme pool in SCD and can inform the development of new targeted therapies.

### Scavenger protein saturation drives the divergent compartmentalization of plasma heme

Our findings reveal a complex dysregulation of heme compartmentalization and clearance in SCD patients (Fig 1a), despite similar total plasma heme concentrations compared to controls (Fig 1b). High levels of reactive heme species in SCD indicate significantly increased deleterious potential that is not captured by total heme alone. Low levels of Hp and Hpx in SCD (Fig 1g, 1h) confirmed an overload of the protective scavenger system due to chronic hemolysis [13]. Hp depletion limits Hp–Hb complex formation (Fig 1f), leading to accumulation of unbound CFH in plasma (Fig 1d). In controls, sufficient Hp ensures efficient CFH neutralization, whereas in SCD, total heme correlates with decreased Hp–Hb and increased CFH, confirming impaired clearance under hemolytic stress (Fig 2). A strong correlation between Hp and Hp-Hb in SCD suggested that the capacity to form Hp-Hb was dependent on the limited availability of Hp (Fig 3a).

Similarly, Hpx exhaustion in SCD leads to accumulation of labile HAH (Fig 3b). HAH comprises heme associated with plasma proteins with a lower affinity than Hpx, such as albumin, alpha-1-microglobulin, or lipoproteins. Hpx–heme complexes were proportionally elevated in SCD but remained minor compared to Hp–Hb (Fig 1e, f), reflecting limited buffering capacity once Hpx is saturated. The lack of correlation between Hpx and Hpx–heme in SCD (S3b Fig) suggests distinct dynamics influenced by hemolysis and clearance mechanisms [21]. Hpx synthesis is induced by heme and inflammation, as reviewed by Tolosano et al. [22], while levels of Hpx-heme depend simultaneously on the concurrent rate of hemolysis and on clearance by hepatocytes and macrophages.

### Plasma heme species are linked to SCD pathophysiology

Excess CFH and heme in SCD activate the innate immune system and endothelium, promoting an inflammatory, pro-adhesive state that contributes to vaso-occlusive crises (VOC), acute chest syndrome (ACS), and long-term organ damage. These complications drive morbidity and mortality in SCD. To explore the relationship between plasma heme species and specific disease processes, we examined their correlations with a comprehensive panel of hematologic, inflammatory, and hemostatic biomarkers.

Our results revealed clearly distinct correlation patterns for different plasma heme species, suggesting associations with specific pathophysiological processes (Fig 4). HAH showed strong correlations with soluble VCAM-1, E-selectin (CD62E), CD40L, and monocyte counts, consistent with biomarker profiles linked to endothelial activation, platelet stimulation, and monocyte recruitment. The endothelial surface expression of adhesion molecules such as VCAM-1 and E-selectin facilitates leukocyte, platelet, and RBC attachment [23,24] driving microvascular occlusion and VOC [25]. Compared to CFH and total plasma heme, HAH exhibited stronger associations with biomarkers linked to VOC, ACS, and organ damage. This highlights the relevance of HAH in characterizing heme‑mediated toxicity in SCD and supports further investigation of therapeutic approaches aimed at enhancing heme scavenging capacity [7,26,27].

CFH (and total plasma heme, (Fig 1a)), correlated with RBC indices (MCV, MCH) and renal function markers (EGF, creatinine). Larger RBC size and higher Hb content could reflect a compensatory reticulocytosis response to increased levels of CFH or the effects of hydroxyurea treatment. The association of CFH with EGF and creatinine suggests acute renal stress from heme-mediated oxidative damage, even in patients without prior renal complications (72% of the cohort, S1 Table). CFH also correlated with hemolytic markers such as aspartate aminotransferase (ASAT) and direct bilirubin, consistent with its origin from RBC breakdown and hepatic stress.

Neither Hp–Hb nor Hpx–heme correlated with pro-inflammatory biomarkers, confirming their inert nature compared to labile heme species. The negative association between Hp-Hb and total heme (Fig 2a) was reflected in partly inverted correlation patterns for these two compartments (Fig 4). Hp–Hb showed positive associations with fibrinogen and thrombin time, suggesting a link to more stable coagulation profiles and reduced fibrinogen consumption. Since Hp–Hb formation depends on Hp availability (Figs 3a, 4), higher Hp–Hb likely reflects better CFH neutralization and reduced coagulation activation.

In contrast, Hpx–heme did not correlate with Hpx levels in SCD (S3b Fig) but was positively associated with total plasma heme (Fig 2). However, there was no common correlation with hemolytic biomarkers between Hpx-heme and reactive heme species, except for hematocrit (Fig 4). Our Hpx assay quantifies both unbound Hpx and Hpx-heme complex. Higher Hpx-heme concentrations may therefore indicate lower levels of unbound Hpx [27,28]. Elevated endothelial and immune activation markers (BAFF, ICAM-1, CCL20, TNFα, TF III, Angiopoietin-2) may reflect reduced protection by unbound Hpx, which has been reported to exert cytoprotective and anti-stasis effects [27] independent of heme clearance [28].

### Total heme assay limitations and the unspecified fraction

The unspecified fraction of total heme represents the difference between the concentration of all quantified heme species and the result from the commercial total heme assay (Fig 1a). This fraction cannot be assigned to a biochemical pool represented by the heme species. However, a strong correlation of unspecified plasma heme with bilirubin in SCD (S2 Fig) may indicate interference of the colorimetric assay with bilirubin and potentially other non-heme chromophores, as found in other studies [20]. In contrast, the methods used to quantify HAH, CFH, Hp-Hb, and Hpx-heme include chromatography separation steps that minimize confounding matrix effects. We speculate that total heme concentrations, and therefore the unspecified proportion of total heme, might be overestimated at high levels of bilirubin, particularly in the SCD group. Although uncertainty remains regarding the unspecified fraction of total heme, our conclusions on divergent compartmentalization are not compromised by this uncertainty, as they are grounded in the direct quantification and proportions of the plasma heme species (HAH, CFH, Hp-Hb, and Hpx-heme). In SCD, total heme, Hpx, and CFH correlated with HAH, whereas no such correlations were observed in controls (Figs 2a, 3b, 4, S3a). This underscores the limitations of these conventional parameters as markers of heme burden, as their concentrations depend not only on hemolysis but also on the saturation state of the heme‑scavenging system. In contrast, HAH directly quantifies labile heme available to exert toxic effects.

### SCD genotype- and treatment effects on heme compartmentalization

We did not observe differences in heme species concentrations between SCD genotypes or patients with and without hydroxyurea treatment (S1 Fig). Patients with the HbSS genotype had higher levels of total plasma heme, which could suggest higher hemolytic activity or reduced plasma heme clearance compared to S/β-thalassemia. However, concentrations of the reactive heme species HAH and CFH were not significantly elevated in HbSS, suggesting similar heme load among genotypes. While potential genotype‑ and treatment‑related effects warrant investigation in larger SCD cohorts, our findings highlight the importance of quantifying labile heme species directly rather than relying solely on total plasma heme.

## Limitations

The cross-sectional design of the study only provides data from a single time point, which limits the ability to infer causality or track temporal changes in heme metabolism and biomarker dynamics. Potential confounders, such as treatment status, disease severity, and comorbidities, may not have been fully assessed and controlled for. Heme levels in whole blood may exceed the concentrations assessed from plasma in this study, as heme readily interacts with proteins, lipids, and cells after CFH breakdown [29]. This study could not quantify the proportion of heme that did not remain in plasma. However, our results show that heme species with reactive potential are more abundant in SCD compared to controls. We speculate that non-plasma heme might also be higher in SCD patients than in controls. This aligns with elevated hemolytic and inflammatory biomarkers, indicating heme toxicity in SCD. Our method for quantifying HAH requires Hpx as a reagent and may not be easily applicable in a clinical setting. Different characteristics (specificity and sensitivity) of the assays used to quantify total plasma heme and the heme species may have introduced bias in comparing absolute concentrations of different heme compartments and in determining the unspecified fraction of the heme pool. Pre-analytical handling can cause RBC lysis, seen as red discoloration in plasma samples from both groups. This may affect some marker levels, but likely did not introduce bias since processing was the same for all. RBC lysis might elevate Hp-Hb complex concentrations in controls, so reference values need future confirmation.

## Conclusion

We provide a comprehensive analysis of plasma heme compartmentalization in SCD, showing that similar total heme levels can mask a profound shift toward reactive heme species when scavenger proteins are depleted. Characterizing individual plasma heme species emerges as a promising approach to better understand heme toxicity in SCD. The strong correlation of an adverse biomarker profile with HAH, and its lack of correlation with the Hpx-heme complex, strengthens the rationale for therapeutic strategies aimed at restoring heme scavenging capacity or enhancing heme clearance. Future studies should test whether heme-compartment-specific metrics can refine risk stratification and guide targeted interventions aimed at mitigating hemolysis‑driven vascular and inflammatory injury in SCD.

## Supporting information

S1 Fig Heme species concentrations by SCD genotype and hydroxyurea treatment. Total plasma heme levels, and concentrations of Hpx-accessible heme (HAH), cell-free hemoglobin (CFH), Hpx-heme complex (Hpx-heme) and Hp-hemoglobin complex (Hp-Hb) are shown for SCD patients with HbS/β‑thalassemia and HbSS genotypes, and for individuals not currently treated with hydroxyurea (no) and for those under treatment with hydroxyurea (yes). Statistical differences between groups are indicated by * (p ≤ 0.05) and NS. (not significant).
(TIF)

S2 Fig Correlation between bilirubin and the unspecified fraction of total plasma heme. Concentrations of total bilirubin are correlated with unspecified fractions of the plasma heme pool in SCD patients and controls (HC). Correlation coefficients (R) and p-values are indicated. Lines show linear regression fits.
(TIF)

S3 Fig Associations between concentrations of plasma heme species in SCD and controls. (a) Hpx-accessible heme (HAH) levels were strongly correlated with concentrations of cell-free hemoglobin (CFH) in SCD, but not in the control group (HC). (b) The Hpx-heme complex was not correlated with Hpx levels in SCD and was weakly correlated in controls. Correlation coefficients (R) and p-values are shown. Lines represent linear regression fits.
(TIF)

S1 Table Characteristics and clinical information of SCD patients collected up to 2 years prior to study inclusion. Data from 36 SCD patients are summarized. The table reports the numbers and percentages of patients with HbSS or S/β‑thalassemia genotypes, those receiving hydroxyurea therapy, and those with documented prior clinical complications.
(DOCX)

S2 Table Clinical hematological parameters in healthy controls and SCD patients. Hematological markers were measured in certified clinical laboratories from plasma, serum, whole blood, and urine. Group means, standard deviations (SD) in brackets are shown. Adjusted p-values reflect statistical comparisons between groups.
(DOCX)

S3 Table Plasma research biomarkers in healthy controls and SCD patients. Protein biomarkers were measured in the plasma of healthy controls and SCD patients. Group means, standard deviations (SD) in brackets are shown. Adjusted p-values reflect statistical comparisons between groups.
(DOCX)

S4 Table Urine research biomarkers in healthy controls and SCD patients. Protein biomarkers were measured in the urine of healthy controls and SCD patients. Group means, standard deviations (SD) in brackets are shown. Adjusted p-values reflect statistical comparisons between groups.
(DOCX)
