# Supplementary material for: Plasma heme pool compartmentalization is linked to pathophysiology in Sickle Cell Disease
Source: PLoS One. 2026 Mar 26;21(3):e0343527. doi: 10.1371/journal.pone.0343527 (PMC13020781; doi:10.1371/journal.pone.0343527)
Supplement: S1 Table — Data from 36 SCD patients are summarized. The table reports the numbers and percentages of patients with HbSS or S/β‑thalassemia genotypes, those receiving hydroxyurea therapy, and those with documented prior clinical complications. (DOCX) [file pone.0343527.s004.docx]

| Characteristic | N = 36 |
| --- | --- |
| **SCD genotype** |  |
| HbSS | 29 (81%) |
| HbSβ⁰^/+^ thalassemia | 7 (19%) |
| **Current treatment with hydroxyurea** | 28 (78%) |
| **Complications in the previous 2 years** |  |
| Vaso-occlusive pain crisis (VOC) | 30 (83%) |
| Hospitalization in the last 2 years | 19 (53%) |
| Organ damage | 32 (89%) |
| Acute chest syndrome | 20 (56%) |
| Acute painful episodes and chronic pain | 31 (86%) |
| Avascular necrosis and osteomyelitis | 16 (44%) |
| Cardiomyopathy and heart failure | 5 (14%) |
| Neurologic complications | 12 (33%) |
| Pulmonary hypertension | 2 (5.6%) |
| Renal complications | 10 (28%) |
| Retinopathy | 11 (31%) |
