# Supplementary material for: Plasma heme pool compartmentalization is linked to pathophysiology in Sickle Cell Disease
Source: PLoS One. 2026 Mar 26;21(3):e0343527. doi: 10.1371/journal.pone.0343527 (PMC13020781; doi:10.1371/journal.pone.0343527)
Supplement: S2 Table — Hematological markers were measured in certified clinical laboratories from plasma, serum, whole blood, and urine. Group means, standard deviations (SD) in brackets are shown. Adjusted p-values reflect statistical comparisons between groups. (DOCX) [file pone.0343527.s005.docx]

| **Analyte** | **Matrix** | **Unit** | **Control Mean (SD)** | **SCD Mean (SD)** | **p-value** |
| --- | --- | --- | --- | --- | --- |
| Iron | Plasma | µmol/L | 17.1 (7.9) | 20.3 (8.1) | 0.160 |
| Transferrin | Plasma | g/L | 2.7 (0.50) | 2.1 (0.34) | < 0.001 |
| Haptoglobin (clin.) | Serum | g/L | 0.99 (0.54) | 0.12 (0.12) | < 0.001 |
| Ferritin | Serum | µg/L | 89.2 (96.9) | 586.8 (1135.1) | 0.042 |
| Bilirubin direct | Plasma | µmol/L | 3.4 (1.7) | 9.2 (3.2) | < 0.001 |
| Bilirubin total | Plasma | µmol/L | 8.8 (5.8) | 35.6 (22.1) | < 0.001 |
| ASAT (aspartate aminotransferase) | Plasma | U/L | 21.8 (7.3) | 36.6 (14.1) | < 0.001 |
| ALAT (alanine aminotransferase) | Plasma | U/L | 20.7 (11.1) | 22.9 (10.6) | 0.359 |
| Creatinine | Plasma | µmol/L | 73.2 (13.8) | 62.2 (25.2) | 0.062 |
| LDH (lactate dehydrogenase) | Plasma | U/L | 196 (33.1) | 406 (126.9) | < 0.001 |
| D-Dimer | Plasma | µg/L | 253 (160) | 1721 (1504) | < 0.001 |
| TAT (thrombin–antithrombin complex) | Plasma | µg/L | 4.6 (2.7) | 6.1 (4.8) | 0.195 |
| INR venous (international normalized ratio) | Plasma | [ratio] | 1.1 (0.05) | 1.1 (0.33) | 0.25 |
| Quick venous | Plasma | % | 87.6 (9.9) | 82.6 (17.4) | 0.195 |
| PT venous (prothrombin time) | Plasma | sec | 11.7 (1.0) | 12.1 (3.3) | 0.532 |
| aPTT (activated partial thromboplastin time) | Plasma | sec | 29.9 (2.9) | 30.9 (5.2) | 0.32 |
| Thrombin time | Plasma | sec | 16.3 (0.99) | 15.6 (1.54) | 0.079 |
| Fibrinogen Clauss manual | Plasma | g/L | 2.5 (0.52) | 2.8 (0.83) | 0.187 |
| Hematocrit | Blood | L/L | 0.41 (0.05) | 1.07 (4.79) | 0.496 |
| Hemoglobin | Blood | g/L | 137.2 (18.5) | 93.4 (14.1) | < 0.001 |
| Leukocytes | Blood | G/L | 5.4 (2.1) | 6.9 (2.5) | 0.042 |
| Thrombocytes | Blood | G/L | 271 (80.0) | 338 (148.1) | 0.047 |
| Erythrocytes | Blood | T/L | 4.8 (0.53) | 3.2 (0.73) | < 0.001 |
| MCV (mean corpuscular volume) | Blood | fL | 84.2 (5.4) | 88.1 (10.7) | 0.134 |
| MCH (mean corpuscular hemoglobin) | Blood | pg | 28.4 (2.5) | 30.3 (4.2) | 0.078 |
| MCHC (mean corpuscular hemoglobin concentration) | Blood | g/L | 338 (13.3) | 344 (13.0) | 0.078 |
| RDW (red cell distribution width) | Blood | % | 13.4 (1.5) | 17.9 (3.3) | < 0.001 |
| MPV (mean platelet volume) | Blood | fL | 10.5 (1.0) | 10.3 (1.0) | 0.59 |
| IPF (immature platelet fraction) | Blood | % | 5.0 (3.2) | 3.8 (3.7) | 0.291 |
| Normoblasts | Blood | /100 Leuc. | 0.00 (0.02) | 2.71 (5.50) | 0.017 |
| Neutrophils | Blood | G/L | 2.9 (1.7) | 3.9 (2.2) | 0.091 |
| Eosinophiles | Blood | G/L | 0.13 (0.09) | 0.17 (0.20) | 0.318 |
| Monocytes | Blood | G/L | 0.45 (0.15) | 0.67 (0.35) | 0.009 |
| Lymphocytes | Blood | G/L | 1.8 (0.60) | 2.0 (0.72) | 0.308 |
| Immature Granulocytes | Blood | G/L | 0.02 (0.01) | 0.03 (0.02) | 0.056 |
| Reticulocytes | Blood | % | 1.4 (0.44) | 7.6 (6.38) | < 0.001 |
| Albumin in urine | Urine | mg/L | 17557 (21692) | 117409 (207115) | 0.032 |
| Protein in urine | Urine | g/L | 0.06 (0.02) | 0.27 (0.71) | 0.183 |
| Creatinine in urine | Urine | µmol/L | 9630 (7462) | 8236 (4114) | 0.407 |
