# Supplementary material for: Plasma heme pool compartmentalization is linked to pathophysiology in Sickle Cell Disease
Source: PLoS One. 2026 Mar 26;21(3):e0343527. doi: 10.1371/journal.pone.0343527 (PMC13020781; doi:10.1371/journal.pone.0343527)
Supplement: S3 Table — Protein biomarkers were measured in the plasma of healthy controls and SCD patients. Group means, standard deviations (SD) in brackets are shown. Adjusted p-values reflect statistical comparisons between groups. (DOCX) [file pone.0343527.s006.docx]

| Analyte | Analyte full name | Unit | Control Mean (SD) | SCD Mean (SD) | p-value |
| --- | --- | --- | --- | --- | --- |
| Angiopoietin-2 | Angiopoietin-2 | pg/mL | 2304 (1168) | 3890 (1936) | 0.001 |
| MCP-1 | Monocyte Chemoattractant Protein-1 | pg/mL | 204 (260) | 198 (195) | 0.988 |
| CD40L | Soluble CD40 ligand | pg/mL | 807 (514) | 1050 (271) | 0.042 |
| EMMPRIN | Extracellular matrix metalloproteinase inducer | pg/mL | 3069 (922) | 4081 (2550) | 0.056 |
| GDF-15 | Growth Differentiation Factor 15 | pg/mL | 271 (81.5) | 1311 (2036.2) | 0.008 |
| ICAM-1 | Intercellular Adhesion Molecule-1 | pg/mL | 240609 (159832) | 168592 (125805) | 0.078 |
| CD62E | Soluble E-Selectin | pg/mL | 23307 (8126) | 38493 (16041) | < 0.001 |
| PlGF | Placental Growth Factor | pg/mL | 18.4 (13.9) | 29.4 (21.3) | 0.047 |
| TNF RI | Tumor necrosis factor receptor 1 | pg/mL | 672 (200) | 944 (791) | 0.131 |
| VCAM-1 | Vascular cell adhesion molecule 1 | pg/mL | 578648 (263859) | 2264630 (927203) | < 0.001 |
| BAFF | B-cell activating factor | pg/mL | 531 (106) | 613 (362) | 0.291 |
| CCL20 | Macrophage Inflammatory Protein-3 alpha | pg/mL | 92.2 (45.5) | 137.2 (112.1) | 0.078 |
| TF_III | Coagulation Factor III/Tissue Factor | pg/mL | 38.7 (17.0) | 41.3 (23.7) | 0.638 |
| Endoglin | Endoglin | pg/mL | 2359 (1837) | 2266 (1529) | 0.706 |
| HGF | Hepatocyte Growth Factor | pg/mL | 136 (159.6) | 199 (74.4) | 0.067 |
| IL-1a | Interleukin-1 alpha | pg/mL | 37.3 (40.3) | 42.5 (56.8) | 0.672 |
| RAGE | Soluble receptor for advanced glycation end-products | pg/mL | 2085 (952) | 1475 (529) | 0.005 |
| CD62P | Soluble P-Selectin | pg/mL | 19168 (5168) | 23407 (7162) | 0.025 |
| uPAR | Soluble urokinase plasminogen activator receptor | pg/mL | 1387 (978) | 1900 (1027) | 0.074 |
| vWF-A2 | Von Willebrand factor (A2 domain) | pg/mL | 878 (555) | 1283 (888) | 0.065 |
| CD62L | Soluble L-Selectin | pg/mL | 574445 (180022) | 581314 (128975) | 0.971 |
| GM-CSF | Granulocyte-macrophage colony-stimulating factor | pg/mL | 3.1 (1.4) | 3.8 (1.6) | 0.091 |
| IL-1b | Interleukin-1 beta | pg/mL | 1.2 (0.80) | 1.8 (3.39) | 0.448 |
| IL-2 | Interleukin-2 | pg/mL | 1.6 (1.2) | 1.3 (1.0) | 0.291 |
| IL-6 | Interleukin-6 | pg/mL | 1.2 (0.79) | 1.6 (0.94) | 0.13 |
| IL-8 | Interleukin-8 | pg/mL | 3.2 (1.4) | 5.7 (3.3) | 0.001 |
| IL-10 | Interleukin-10 | pg/mL | 1.3 (0.71) | 1.6 (0.60) | 0.078 |
| TNFa | Tumor necrosis factor alpha | pg/mL | 10.6 (9.8) | 12.0 (3.9) | 0.359 |
| VEGF | Vascular Endothelial Growth Factor | pg/mL | 9.0 (4.2) | 15.1 (13.3) | 0.042 |
| HMGB1 | High-Mobility-Group Box 1 | ng/mL | 1.9 (1.0) | 3.3 (6.9) | 0.32 |
| MD-2 | Myeloid differentiation factor-2 | ng/mL | 162 (402) | 499 (1507) | 0.291 |
