# Supplementary material for: Plasma heme pool compartmentalization is linked to pathophysiology in Sickle Cell Disease
Source: PLoS One. 2026 Mar 26;21(3):e0343527. doi: 10.1371/journal.pone.0343527 (PMC13020781; doi:10.1371/journal.pone.0343527)
Supplement: S4 Table — Protein biomarkers were measured in the urine of healthy controls and SCD patients. Group means, standard deviations (SD) in brackets are shown. Adjusted p-values reflect statistical comparisons between groups. (DOCX) [file pone.0343527.s007.docx]

| Analyte | Analyte full name | Unit | Control Mean (SD) | SCD Mean (SD) | p-value |
| --- | --- | --- | --- | --- | --- |
| Calbindin | Calbindin | ng/mL | 8.2 (11.6) | 5.6 (5.8) | 0.291 |
| KIM-1 | Kidney Injury Molecule-1 | ng/mL | 0.57 (0.71) | 0.56 (0.56) | 0.961 |
| TIMP-1 | Tissue inhibitor of metalloproteinases-1 | ng/mL | 4.1 (6.2) | 4.1 (5.1) | 0.876 |
| TFF-3 | Trefoil Factor 3 | ng/mL | 28.1 (30.4) | 37.0 (59.0) | 0.577 |
| GSTa | Glutathione S-transferase alpha | ng/mL | 10.8 (27.9) | 12.0 (20.6) | 0.738 |
| FABP-1 | Fatty Acid Binding Protein 1 | ng/mL | 7.3 (4.4) | 9.5 (4.7) | 0.102 |
| Collagen IV | Urinary type IV collagen | ng/mL | 21.4 (17.4) | 19.1 (16.0) | 0.672 |
| NGAL | Neutrophil Gelatinase-Associated Lipocalin | ng/mL | 46.3 (67.2) | 106.9 (188.1) | 0.135 |
| EGF | Urinary Epidermal Growth Factor | ng/mL | 19.7 (18.3) | 18.7 (12.7) | 0.848 |
| Clusterin | Urinary Clusterin | ng/mL | 2484 (3075) | 2448 (4514) | 0.971 |
| OPN | Osteopontin | ng/mL | 952 (992) | 698 (549) | 0.285 |
| Cystatin C | Urinary cystatin C | ng/mL | 19.8 (25.7) | 28.8 (62.6) | 0.521 |
| a1-Microglobulin | Urinary alpha-1 microglobulin | ng/mL | 338 (345) | 1462 (1357) | < 0.001 |
| NAG | N-Acetyl-β-D-glucosaminidase | U/L | 1.8 (1.8) | 3.6 (3.6) | 0.037 |
| MCP-1 | Urinary monocyte chemoattractant protein-1 | pg/mL | 204 (260) | 198 (195) | 0.988 |
